# Development of an immunoblotting assay for serodiagnosis of *Burkholderia mallei* infection: the whole-cell proteome-based paradigm

**Published:** 2019-06

**Authors:** Sajjad Yazdansetad, Nader Mosavari, Keyvan Tadayon, Iraj Mehregan

**Affiliations:** 1 Department of Biology, Science and Research Branch, Islamic Azad University, Tehran, Iran; 2 Department of Tuberculin and Mallein, Razi Vaccine and Serum Research Institute, Agricultural Research, Education and Extension Organization (AREEO), Karaj, Iran; 3 Department of Veterinary Aerobic Bacterial Vaccines, Razi Vaccine and Serum Research Institute, Agricultural Research, Education and Extension Organization (AREEO), Karaj, Iran

**Keywords:** *Burkholderia mallei*, Immunoblotting, Glanders, Whole-cell proteome, Serodiagnosis

## Abstract

**Background and Objectives:**

*Burkholderia mallei* is the leading cause of glanders, a highly transmittable and an OIE-notifiable disease of equidae. Despite the importance of *B. mallei*, little is known about serodiagnosis of glanders. The present study aimed to develop an immunoblotting assay based on whole-cell proteome of *B. mallei* to enable accurate serodiagnosis of glanders.

**Materials and Methods:**

Three farm horses were subcutaneously immunized with a crude suspension (10^6^ cfu/ml) of heat-inactivated *B. mallei* formulated with incomplete Freund’s adjuvant (IFA) to achieve a hyperimmune sera panel. The immunization was done for 1, 14 and 28 days with 1 dose of 1 ml antigen containing 10^6^ cfu/ml. The hyperimmunity of sera was confirmed by CFT. *B. mallei* whole-cell proteome was prepared through sonication and the protein content was visualized by SDS-PAGE and quantified by Western blot using HRP-conjugated rabbit anti-horse IgG. A comprehensive set of positive and negative horse sera validated the test.

**Results:**

A ladder pattern of the *B. mallei* immunoreactive antigens was seen within the region of 20–90 kDa clearly and the immunoblot was scored positive, while no reaction was seen for the negative sera. The Western blot assay indicated a noticeably higher diagnostic specificity for positive or negative sera of glanders.

**Conclusion:**

The whole-cell proteome-based immunoblot proved reliable and straightforward in our study. The prepared antigen was adaptable for application in immunoblotting. We assumed this improved immunoblotting system provides appropriate sensitivity and also specificity expected in serodiagnosis of glanders in endemic areas and typically in less-developed countries.

## INTRODUCTION

*Burkholderia mallei*, a Gram-negative bacillus, is the causative agent of the febrile disease known as glanders, a pulmonary disease, and a facultative intracellular pathogen mostly of equidae (1, 2). Glanders is a highly contagious and zoonotic disease primarily affecting solipeds (i.e., horses, mules, and donkeys) (3). Generally, glanders in horse tends to be a chronic disease but in other equidae (i.e., mule, zebra, and donkey) the disease has an acute nature (3, 4). In equids, glanders is transmitted by oro-nasal mucous membrane invasion and inhalation routes (5). The common symptoms of glanders include nasal discharge (mucopurulent), lung lesions and nodules involving the liver and spleen (3).

Humans are accidental hosts for glanders and naturally acquired glanders is sporadic. Veterinarians, farmers, and horsekeepers are professionals who are most likely to acquire the disease through their likely close proximity to diseased animals (1, 4). In humans, *B. mallei* can be directly transmitted by invasion of nasal, conjunctival and oral mucous membranes; abraded or lacerated skin and by inhalation into the lungs. The manifestations correlate with the route of infection and the disease can be either of an acute or chronic nature (4). Human glanders cases have previously been reported from Austria, Cameroon, Curacao, Sri Lanka, Turkey and the USA, but human epidemic has not been recorded (3). The once globally scattered disease is now endemic mostly in Africa, Asia, Middle East, as well as Central and South America (2, 6). The disease recently qualified as re-emergent due to the increased number of cases reported in several parts of the world during the last 20 years (3). Glanders has remained in Iran over more than 60 years by having neighboring countries located in the endemic disease focus (7).

*B. mallei* has many intrinsic antibiotic resistances (e.g., earlier beta-lactams, colistin, fluoroquinolones, macrolides and aminoglycosides) (8). There is no globally-accepted chemical treatment regimen in human cases while suspicious/diseased animals are normally euthanized fearing dissemination of the disease (4). Glanders has a case fatality rate of 95% in untreated humans with septicaemia and 50% even after administration of antibiotic therapy (9). *B. mallei* is a category B classified biothreat agent by Center for Disease Control (CDC) and a listed pathogen by Biological Weapons Convention on the grounds of its flexibility to be weaponized (4, 10). In fact, *B. mallei* was among the few biological agents used in the World Wars I and II and is one of the first examples of the use of bacteria in warfare (2, 3, 11). The severe and debilitating diseases caused by these intracellular pathogens are difficult to diagnose, and there are currently no commercially available vaccines for either human or veterinary use (10).

The traditional method of diagnosis of glanders is the mallein intradermal test where a small volume of *B. mallei* culture extract (e.g. 0.1 ml) is administrated into the lower eyelid of the suspected equid and reactions are assayed after specific time (12). Poor efficiency and indecisive results in acute and progressed cases of the disease along with cross-reactivity with the other serological tests seriously lower the clinical performance of this test (13). The serological tests, including complement fixation test (CFT), enzyme-linked immunosorbent assays (ELISA), Rose Bengal plate agglutination test (RBPT), indirect fluorescent antibody test (IFAT), indirect hemagglutination test (IHAT), and counter immunoelectrophoresis test (CIET) have been used for diagnosis of glanders where there is technically no flawless method of choice (14). CFT however, is currently the most widely used test in international trade of equines and is recommended by the World Organisation of Animal Health (OIE) (12) though some limitations such as false positive/negative results have been noticed (5, 15, 16). The CFT is a labor-intensive and time-consuming test with sensitivity of 90–95%, and a questionable specificity (5). There is, therefore, room for development of an immunoblotting-based method providing high specificity. Given that the present study was conducted to assess suitability of immunodominant protein antigens screened from whole-cell protein content of *B. mallei* for glanders serodiagnosis. Technical issues in preparation, selection, extraction and characterization of whole-cell protein repertoire obtained from Iranian isolates of the bacterium were addressed.

## MATERIALS AND METHODS

### Bacterial strains, growth conditions, and antigen preparation.

The smooth, virulent laboratory strain of *B. mallei* Razi 325 (Razi Type Culture Collection; RTCC 2375) was used as a control strain. This non-native strain has been employed for the production line of the mallein at the Razi Vaccine and Serum Research Institute, IR, Iran and was kindly provided by the Swedish cooperative laboratory in 1956. Furthermore, we have isolated 4 new strains of *B. mallei* (Tiger, isolated from tiger in 2010; Kordan, isolated from mare in 2013; Oshnavieh, isolated from mule in 2014; and Semirom, isolated from mare in 2016) regarding the endemic disease in Iran over a 7-year period ranging from 2010 to 2016. Considering the Biosafety Level 3 (BSL-3) facility, the *B. mallei* strains were aerobically cultured in nutrient broth supplemented with glycerol 4% for 48 h at 37°C. Whole-cell proteome of bacterial isolates was extracted as follow: concisely, the culture pellet was centrifugation at 9,000 × g for 15 min at 4°C then washed thrice with ice-cold phosphate buffered saline (0.01 M PBS, pH 7.2). The pellet was resuspended in PBS containing 1 mM phenylmethane sulfonyl fluoride (PMSF) and sonicated on ice at 90 amplitude for 5 cycles of 1 min pulsing with 1 min rests between the pulses (Hielscher-Ultrasound Technology, PN-66-NNN, Germany) then centrifuged at 9000 × g for 10 min. The supernatant was treated with 1 ml lysis buffer (Urea 8 M, Tris-Hcl 10 mM, NaH_2_POH_4_ 100 mM, EDTA 1 M, SDS 1%). The supernatant was precipitated with tricarboxylic acid (TCA) (Merck, Germany). The washed pellet was resuspended in PBS (0.01 M, pH 7.2) (5). Total protein concentration was evaluated using the Bradford method by measuring the absorbance at 595 nm (UV/Vis spectrophotometer 6320D, Jenway, UK) using bovine serum albumin (BSA) as the standard.

### Hyperimmune sera production in rabbit and horse.

A male albino rabbit with a weight of about 1.5 kg and 6 months old was immunized with a 10^6^ cfu/ml crude suspension of heat-inactivated *B. mallei*. The same condition was applied for *Pseudomonas aeruginosa* ATCC^®^ 27853 and *Burkholderia pseudomallei* ATCC^®^ 23343 as the control strains. The immunization was done intracutaneously with 10 doses of 0.5 ml antigen containing 10^6^ cfu/ml in three days of 0, 3 and 7. The next step immunization was continued in days of 14, 21, 28, 35 and 42 in the ear marginal vein by 0.5 ml antigen as described above. The hyperimmune serum was obtained 7 weeks followed by the first immunization.

The immunization of 3 horses was subcutaneously carried out with a crude suspension (10^6^ cfu/ml) of heat-inactivated *B. mallei* formulated with incomplete Freund’s adjuvant (IFA) (InvivoGen, USA) to achieve a hyperimmune sera panel. The immunization was done for 1, 14, 28 days with 1 dose of 1 ml antigen containing 10^6^ cfu/ml. The titer was parallel analyzed with CFT and Western blot assay parallelly using the homologous antigen twice-weekly and for 10 weeks followed by the first step of immunization (15). *Burkholderia pseudomallei* ATCC^®^ 23343 was used as the control strain. All the animal experiments were authorized by the ethics committee of Razi Vaccine & Serum Research Institute, Karaj, Iran.

### Serum samples collection, enzyme-linked immunosorbent assay (ELISA), and complement fixation test (CFT).

A total of 121 sera samples were obtained from different horse populations in various geographical areas of Iran from 2016 to 2018. The sera samples were parallelly monitored by ELISA and CFT for glanders according to the instructions of the OIE Manual of Diagnostic tests and Vaccines for Terrestrial Animals. Appropriate antigen concentration and serum dilution was optimized by checker-board titration method using glandes positive and negative control sera. Indirect ELISA was launched in 96-well microplates (Corning^®^ Round-bottom ELISA Microplates, Singapore) coated with 10 μg/ml *B. mallei* LPS antigen in 0.05 M carbonate-bicarbonate buffer, pH 9.6 at 4°C overnight. The plates were twice washed with PBS-T, pH 7.2 (0.01 M PBS, 0.05% tween 20) and blocked with 2.5% skimmed milk in PBS-T for 2 h at 37°C. Test sera diluted 1:200 in blocking buffer was appended in duplicate wells for 1 h at 37°C. Then, the wells were washed five times with PBS-T. Rabbit anti-horse (IgG) antibodies conjugated to horseradish peroxidase (HRP) diluted 1:10,000 were added to the wells and incubated for 1 h at 37°C. After washing five times with PBS-T, the plate was developed in the dark for 10 min with TMB (3,3′,5,5′-Tetramethylbenzidine) chromogenic substrate. The reaction was stopped by addition of 2.5 M H_2_SO_4_ and absorbance was measured at 492 nm in an ELISA plate reader (BioTek 800 TS, USA). Cutoff value for indirect ELISA was determined by 15 randomly assigned glanders-negative sera samples according to the above-described method. The cutoff optical density at 492 nm was measured by the addition of 2 standard deviations (SDs) to the mean OD_492_ of the sera samples, and test samples with an OD_492_ greater than the cutoff were preserved as positive.

CFT was carried out with the standard method as described by the OIE. Briefly, equine sera were diluted 1:5 in veronal buffered saline (VBS) (bio-PLUS™, USA) containing 0.1% gelatin. The diluted sera were inactivated for 30 min at 58°C. Twofold dilutions of the sera were prepared using veronal buffer in 96-well round-bottom microtiter plates. Serum, complement, and antigen were gently mixed and incubated for 1 h at 37°C. A 2.5% suspension of sensitized washed sheep red blood cells was added. Plates were incubated for 1 h at 37°C and then centrifuged for 5 min at 600 × g. Serum haemolysis at the 1:5 dilution was considered as negative. The controls of the positive, negative, complement/anti-complement, antigen, and haemolytic system were remarked (12).

### Sodium dodecyl sulphate-polyacrylamide gel electrophoresis (SDS-PAGE).

The protein repertoire of *B. mallei* strains was separated on denaturing 6–12% polyacrylamide gradient gel for 15 h at 25 V according to the Laemmli method. Coomassie brilliant blue G-250 was used for staining and the molecular mass of the proteins was estimated using protein standards.

### Western blotting.

The prepared antigens of the *B. mallei* native strains were equally mixed for further investigation of immunoblotting. A replicate gel was transferred to polyvinylidene difluoride (PVDF) membrane (Roche Mannheim, Germany) for 2 h at 300 V. Prior to use, the membrane was activated by soaking in methanol for 20 seconds. The membrane was blocked with 5% BSA for overnight. After being washed with TBS-T (Tris-HCl 20 mM, NaCl 150 mM, pH 7.5-Tween 0.05%) three times, the membrane was cut into slim strips, then incubated with a 1:200 dilution of equine/rabbit sera as the primary antibody, followed by washed three times with TBS-T, and a 1:10,000 dilution of HRP-conjugated rabbit anti-horse IgG as the secondary antibody (Amersham BioSciences, UK) in TBS-T for 2 h. The signals were followed by chemiluminescent 3,3′-diaminobenzidine (DAB) (Sigma-Aldrich, USA) and H_2_O_2_ as the substrate.

### Statistical analysis.

McNemar’s test by the Prism 8.0 statistics tool was used to interpret of the sensitivity, specificity, and positive and negative predictive values of the immune assay methods.

## RESULTS

### Protein profile of Iranian isolates of *B. mallei*.

**Fig. 1.**
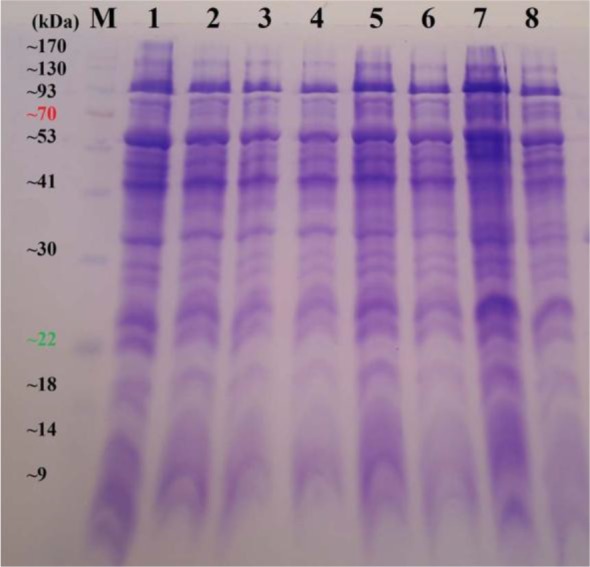
SDS-PAGE analysis of protein profile of Iranian isolates of *B. mallei*. Lane M: Prestained Protein Ladder (Thermo Scientific™, Malaysia); Lanes 1 and 2: *B. mallei* Kordan strain; Lanes 3 and 4: *B. mallei* Semirom strain; Lanes 5 and 6: *B. mallei* Tiger strain; Lanes 7 and 8: *B. mallei* Oshnavieh strain growth. All strains were grown on nutrient broth supplemented with glycerol 4% and glycerol 0.5%, respectively.

Two culture conditions were considered for the *B. mallei* native strain growth. Regarding the glycerol as the key factor on the growth of *B. mallei*, once the bacterial strains were cultured in nutrient broth supplemented with glycerol 0.5% and once again increased with glycerol 4%. No difference was seen at protein expression level by SDS-PAGE analysis in both conditions (Fig. 1). The protein concentration was measured by absorption at 280 nm for each preparation and was estimated at 1.5 mg/ml on average.

### ELISA, CFT and Western blot assay.

**Fig. 2.**
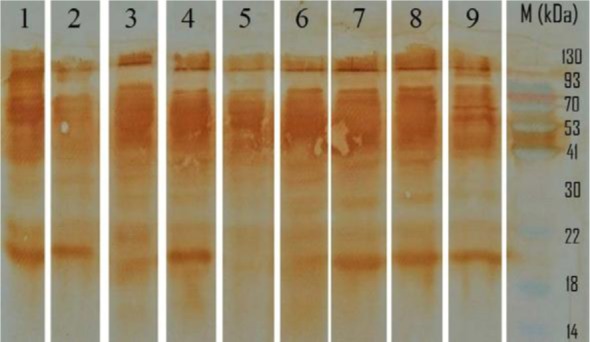
The panel of immunoreactive protein antigens of equine sera samples by Western blot analysis. Lane M: Prestained Protein Ladder (Thermo Scientific™, Malaysia); Lanes 1–9: The true positive sera samples of horses.

**Fig. 3.**
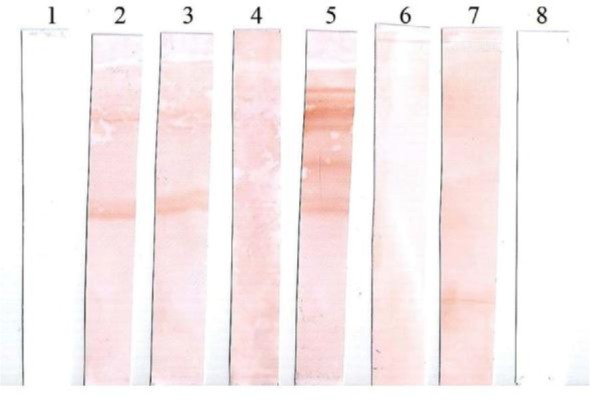
Lanes 1, *P. aeruginosa*-charged rabbit serum, Lane 2: *B. pseudomallei*-charged rabbit serum, Lane 3: *B. pseudomallei*-charged horse serum, Lanes 4, 6, and 7: Negative serum, Lane 5: Positive serum, Lane 8: Negative control.

A total of 13 out of 121 sera samples were positive by indirect ELISA regarding the cutoff OD_492_ (mean OD_492__2 SDs). One false negative and 2 false positive were found by the indirect ELISA. The sensitivity and specificity of indirect ELISA were evaluated (91.66%; 95% CI 89.1–94.3) and (98.21%; 95% CI 95.3–99.0), respectively. The positive predictive value (PPV) and negative predictive value (NPV) were estimated (84.61%; 95% CI 78.3–89.4) and (99.09%; 95% CI 96.4–99.7), respectively. CFT assay indicated that 12 out of 121 sera were positive with sensitivity and specificity of (91.66%; 95% CI 89.1–94.3) and (99.09%; 95% CI 96.4–99.7), respectively. Only 1 false negative and 1 false positive were found by the CFT. The PPV and NPV of CFT were estimated (91.66%; 95% CI 89.1–94.3) and (99.09%; 95% CI 96.4–99.7), respectively. Further, sera with suspicious results in indirect ELISA and anticomplementary activity in CFT were retested by Western blotting. 11 ELISA and CFT-positive sera were assayed by Western blot showing ladder-like bands pattern of immunoreactive proteins and were counted positive. Although, 110 out of 121 sera were scored true negative by the Western blot assay. The immunoproteomic strategy using specific immunoreactive proteins of *B. mallei* was applied for the serodiagnosis of glanders with the high sensitivity and specificity of 100% (95% CI). Furthermore, it seems that the most immunoreactive proteins of *B. mallei* were almost ranged from 20–90 kDa. The charged strips with the equine sera panel containing immunoreactive protein antigens are shown in Fig. 2. *P. aeruginosa* and *B. pseudomallei*-mediated hyperimmune sera produced in albino rabbit showed no detectable bands pattern (Fig. 3).

## DISCUSSION

Here, we describe an immunoblotting method based on the whole-cell proteome of *B. mallei* for serodiagnosis of glanders with a reliable sensitivity and specificity assessment of the immunoreactive proteins. The seroprevalence study was conducted among Iranian horses by using ELISA, CFT and also Western blot with combinations of *B. mallei* native strains. The ELISA and CFT false positives and distrustful sera were re-tested with Western blot and indicated considerable differences in specificity of serodiagnosis by the Western blot in comparison with the ELISA and CFT. Western blot assay confirmed positive and excluded false positive ELISA and CFT results with a 100% sensitivity and specificity. We know that the diagnosis of glanders is highly problematic due to low awareness of the clinical manifestations of disease, nonexistence of experience among microbiologists outside the endemic areas, lack of suitable culture media and identification systems in the custodian laboratories, and the biosafety facilities (10).

In an endemic area such as Iran, early detection of the disease would be helpful for controlling glanders by adopting the test and slaughter policy (3, 7). Currently, there is no effective vaccine against *B. mallei.* Thus, the only successful control option for glanders is testing and isolating positive cases (3). The effectiveness of diagnostic tests depends on their sensitivity and specificity, accuracy, predictive values, and field applicability. However, the efficiency of the tests can significantly improve by local strains for the glanders detection (17). Despite the importance of *B. mallei* as a hazardous zoonotic pathogen and potential bioweapon agent, little is known about the serodiagnosis of glanders. It is noteworthy that the serological tests for RBPT, IHAT, CIET, and IFAT which are available for diagnosing glanders have also deficiencies and unacceptable specificity and/or sensitivity (18). Regarding the OIE recommendation, specific delayed hypersensitivity reaction following intracutaneous application of mallein as well as CFT has been used for the diagnosis of *B. mallei* infection (12, 19). Although CFT is an only available way to stop the establishment of *B. mallei* in niches and has formally been prescribed for the international trade of equidae, it has some shortcomings including insufficient sensitivity and specificity, improper results from sera having anticomplementary features as well as false positive/negative results (5, 15, 16). It has been reported that CFT in serodiagnosis of glanders in donkey, mule, and pregnant mare may give inconclusive results due to the anticomplementary activity of sera (19, 20). CFT may also lead to the destruction of some healthy animals due to the occurrence of false positives (17).

The whole-cell proteome-mediated immunoblotting can be adopted for new diagnostic target antigens existing in the serum. Furthermore, the method is highly specific test for sera with anticomplementary or suspicious reactions in CFT (21). In a recent study, the purified lipopolysaccharide (LPS) of *B. mallei* was made to diagnose positive and negative sera obtained from horses and mules from endemic and non-endemic areas by using the Western blot technique showing a significantly higher diagnostic specificity in comparison with the CFT particularly in glanders-free areas (15). However, many studies have believed that the extraction and purification of LPS from *B. mallei* are complicated, time-consuming, and unsafe for the laboratorians (18). On the other hand, the LPS is a nonspecific antigen having common epitopes in many Gram-negative bacteria (16). *B. mallei* and *B. pseudomallei* have high cross-reactive epitopes in their LPS underlying false positive results in serodiagnosis (22). We assume the whole specific protein antigens assayed by the Western blot can detect the *B. mallei*-specific epitopes as well as genus-specific epitopes.

It is evident that there are difficulties in specific detection and differentiation of *B. mallei* and *B. pseudomallei* due to the derivation probability of *B. mallei* from an ancestral *B. pseudomallei* and having high similarity at their genome level (18). The other perspectives for the immunoserodiagnosis of *B. mallei* and *B. pseudomallei* using well-characterized single antigen by recombinant technology or naturally purified are discussed due to cross-reactivity chance (23). Our finding indicated that the whole specific immunoreactive protein antigens in glanders serodiagnosis overcome this shortcoming and showed no interference between *B. mallei* and *B. pseudomallei*. It is important in international trade of animals which have infections with dangerous zoonotic agents *B. pseudomallei* and/or *B. mallei* (3).

Preventing and controlling programs of glanders have been pointed out to health authorities in Iran (12). The illegal importation of *B. mallei*-infected horses from the neighboring countries can disseminate this disease (24). Moreover, the precious population of horses should be periodically checked for glanders. The healthy carriers (i.e., subclinically infected animals) should also be screened for controlling persistence and propagation of disease (12). The developed Western blot assay can be helpful in serodiagnosis of the healthy and suspicious horses in both glanders free and endemic regions with a high negative likelihood ratio for animal transport, international trade, and preparedness plans.

In conclusion, Western blotting is a highly suggestive confirmatory test along with the CFT to increase the serodiagnosis specificity of glanders. So, it can be scheduled as a trustworthy early serodiagnosis test in OIE Manual for eradication programs.
